# Shear Capacity Evaluation of the Recycled Concrete Beam

**DOI:** 10.3390/ma15103693

**Published:** 2022-05-21

**Authors:** Qiuwei Yang, Xi Peng, Yun Sun

**Affiliations:** 1 School of Civil and Transportation Engineering, Ningbo University of Technology, Ningbo 315211, China; sunyun@nbut.edu.cn; 2 Engineering Research Center of Industrial Construction in Civil Engineering of Zhejiang, Ningbo University of Technology, Ningbo 315048, China

**Keywords:** recycled aggregate concrete, beam structure, shear capacity, shear fracture, size effect

## Abstract

Compared with traditional concrete beams, recycled concrete beams are more prone to cracking and shear failure. Generally, shear failure is a brittle failure and its failure consequences are often very serious. Thus, the shear capacity is an important parameter in the design and testing for beam structures. In this work, the computation method and size effect on shear capacity of recycled concrete beams without stirrups are studied. Four recycled aggregate concrete beams with different sizes are tested by the bending experiment to obtain their ultimate shear capacities. By keeping the shear span ratio unchanged, the variation laws of mechanical parameters such as cracking load, ultimate shear capacity and shear strength for these beam specimens are studied. From the experiment results, it is concluded that the shear capacities of beams with lengths of 740 mm, 1010 mm, 1280 mm and 1550 mm are 86.3 kN, 106 kN, 124.7 kN and 177.7 kN, respectively. The corresponding shear strengths are 6.84 MPa, 5.59 MPa, 4.9 MPa, and 5.56 MPa, respectively. Nine computation formulas of shear capacity in the literature, such as ACI 318M-14, EN 1992-1-1, GB50010-2010 and so on, are used to calculate the shear capacities of these recycled concrete beams for comparison. The comparative study shows that it is feasible to consider the size effect in the computation of shear capacity for the recycled concrete beam.

## 1. Introduction

Concrete beam is one of the most common components in building and bridge structures. Flexural and shear capacity are the two most important parameters in the design of concrete beam structure. At present, the computation theory of shear capacity of ordinary concrete beams is been relatively mature. Thus, the existing research mainly focus on the shear capacity of high-strength concrete, fiber-reinforced concrete or recycled concrete beams. In recent decades, many researchers have carried out a lot of theoretical and experimental studies in this field. Elzanaty and Nilson [1] carried out the shear strength experiment for the prestressed concrete beams. They found that ACI code equations gave conservative results for the cracking loads of these beams. Salandra and Ahmad [2] tested 16 beams made of lightweight high-strength concrete to obtain the shear capacities. Test results indicate that the predictions by ACI Building Code are unconservative for a few beams. Malek and Saadatmanesh [3] used truss analogy and compression field theory to analysis the shear capacity of reinforced concrete beams. Their analysis results have shown close agreement to experimental results. Li et al. [4] studied the effect of composite carbon fabric shear reinforcement on the ultimate strength of a reinforced concrete beam. They found that the beam shear capacity is not the simple superposition of the concrete shear strength plus the shear strength due to steel stirrups and the composite fabrics. Han et al. [5] studied the shear capacity of reinforced concrete beams with recycled-aggregate. Their results indicate that the ACI Building Code predictions are unconservative for the recycled aggregate concrete beams. Majdzadeh et al. [6] found that fiber reinforcement can enhance the shear load capacity and shear deformation capacity of reinforced concrete beams. Sadati et al. [7] investigated the shear capacity of full-scale reinforced concrete beams fabricated with high volume fly ash and coarse recycled concrete aggregate. Colajanni et al. [8] proposed an estimation model for the shear capacity in reinforced concrete beams with web reinforcement. Lee et al. [9] proposed a shear strength model for steel fiber-reinforced concrete beams. Torres and Ahmadi et al. [10] and Naderpour et al. [11] used the artificial neural network to analyze the shear strength of reinforced concrete beams. Ly et al. [12], Kamgar et al. [13], and Prayogo et al. [14] used artificial intelligence techniques to predict the shear capacity of reinforced concrete beams. By considering size effect, Wang et al. [15] proposed the new computation formula for the shear capacity of reinforced concrete beams without web reinforcement. Bažant [16,17,18] extended the size effect law to calculate the shear capacity of reinforced concrete beams without web reinforcement. Luo [19] further deduced Bažant’s shear strength formula by considering diagonal-tension failure and shear-compression failure. Etxeberria et al. [20] studied the shear properties of recycled concrete beams. They found that the shear performance of the beam will not be affected when the replacement rate of recycled aggregate is less than 25%. The experimental results by Gonzalez et al. [21] show that there is little difference in deflection and shear capacity between recycled concrete beam and ordinary concrete beam. Zhou et al. [22] found that the shear failure process of recycled concrete beams is basically similar to that of ordinary concrete beams. Choi et al. [23] investigated the effect of different recycled aggregate replacement ratio on the beam shear strength. It was found that the current shear strength computation formula can be used for recycled concrete beams if recycled aggregate replacement rate is less than 50%. Zhou et al. [24] proposed a modified formula for calculating the shear capacity of recycled concrete beams. Wu et al. [25] studied the shear properties of recycled concrete beams under different strength grades, different shear span ratios and different stirrup ratios. Fathifazl et al. [26,27] found that the existing shear design methods can be extended to the recycled concrete beams. Zsutty et al. [28] used the statistical fracture mechanics method to calculate the shear capacity of reinforced concrete beams without stirrups. Katkhuda and Shatarat [29] found that both fracture mechanics method and MCTF method are suitable for the computation of recycled concrete beam shear strength. Arezoumandi et al. [30] found that the shear strength of 100% recycled aggregate concrete beam is about 12% lower than that of conventional concrete beam. 

Although much progress has been made in the shear capacity of concrete beams, there have been few reports on the size effect of the shear capacity for the recycled aggregate concrete beam. The innovation of this paper mainly lies in two aspects. The first is to investigate the size effect law of the shear capacity of recycled concrete beams. The variation laws of other related mechanical parameters such as cracking load, the shear fracture strength and shear strength for recycled concrete beams are also investigated simultaneously. The second is to carry out the comparative study of several existing theoretical formulas for the beam shear capacity. The applicability and limitation of each shear capacity formula for recycled concrete beams are investigated from the comparative study. To this end, four recycled concrete beams with different sizes were designed and fabricated in the lab. The bending tests were carried out for these beam specimens to obtain their shear capacity. Several theoretical formulas of shear capacity in the literatures, such as ACI 318M-14, EN 1992-1-1, GB50010-2010 and so on, are used to calculate the shear capacities of these recycled concrete beams for comparison. The test results show that the shear capacity of recycled concrete beams has obvious size effect. It is very necessary to consider the size effect in the shear capacity computation formula for recycled concrete beams. The presentation of this work is organized as follows: Section 2 presents the materials and theoretical methods used in the bending experiment of the recycled concrete beam. Section 3 presents the analysis and comparison between experimental results and theoretical results by nine calculation formulas. Finally, the conclusions of this work are summarized in Section 4.

## *2.* Materials and Methods

### 2.1. Experimental Materials

**Figure 1:**
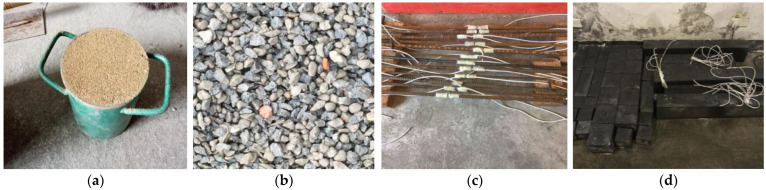
Fine aggregate, recycled coarse aggregate, reinforcement bar, and specimens. (**a**) The fine aggregate; (**b**) The recycled coarse aggregate; (**c**) HRB400 ribbed steel bars; (**d**) Block and beam specimens.

The main materials used in the recycled concrete beams include cement, fine aggregate, recycled coarse aggregate and reinforcement bar. As shown in Figure 1, the fine aggregate is the natural river sand and the recycled coarse aggregate comes from building demolition. The longitudinal reinforcement used at the bottom of beam is HRB400 ribbed steel bar. The tested physical parameters of these materials are presented in Table 1, Table 2 and Table 3, respectively. The used cement is the ordinary PM32.5 Portland cement. Powdered naphthalene superplasticizer is used in the process of mixing concrete. The mix proportion design of the recycled aggregate concrete in this experiment is shown in Table 4. The recycled concrete is casted together with the steel bars by the wood formworks for the four beams. After 28 days of curing, the wood formworks are removed to form the beam specimens. The curing temperature and relative humidity are about (20 ± 2)°C and 95%, respectively.

### 2.2. Shear Capacity Test by Four-Point Bending

**Figure 2:**
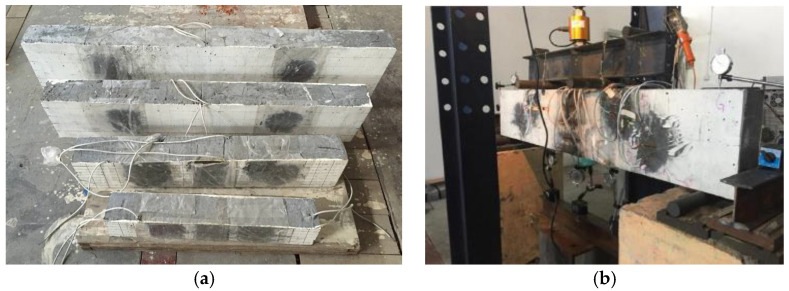
Beam specimens and the loading equipment. (**a**) Beam specimens with different sizes. (**b**) Loading equipment of four-bending test.

**Figure 3:**
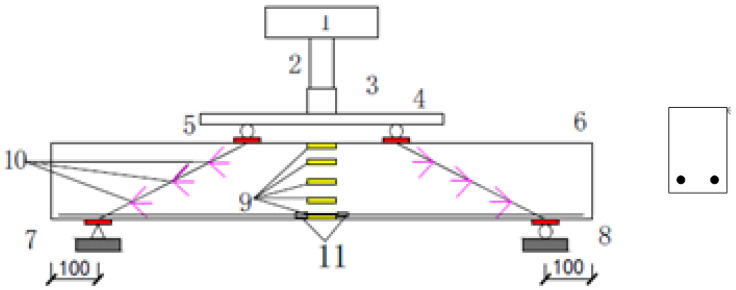
The layout of measuring points: 1 Reaction Frame, 2 Lifting Jack, 3 force sensor, 4 distributive girder, 5 Cushion block, 6 Test-piece, 7 pin support, 8 roller support, 9 Midspan concrete foil strain gauge, 10 Concrete foil strain rosette at shear span, 11 Longitudinal bar foil strain gauge.

As shown in Figure 2, four recycled concrete beams with different sizes are used to investigate the size effect of shear capacity by four-point bending testing. In the experiment, the force sensor measures the load, the strain gauge measures the strain of concrete and reinforcement, and the dial indicator measures the deflection. Figure 3 shows the layout of measuring points for these beam specimens. In Figure 3, note that the distance between the support and the edge of the beam is 100 mm for the four beam specimens. Table 5 presents the beam size, clear span, reinforcement design, and thickness of reinforcement cover. From Table 5, the ratios of cross-section height to span length for the four beams are equal, i.e., $\frac{120}{540}=\frac{180}{810}=\frac{240}{1080}=\frac{300}{1350}$. The purpose of this design is to facilitate the investigation of the size effect of shear capacity for these beam specimens. From the test of blocks shown in Figure 1d, the compressive strength, tensile strength, elastic modulus and Poisson’s ratio of the recycled concrete are $f_{c}^{\prime }=53.4\;$ MPa, $f_{tk}=2.48\;$ MPa, $E=2.588\times 10^{4}$ MPa, and μ=0.1951, respectively. According to the length of the beam, the beam specimen numbers are B-740, B-1010, B-1280, and B-1550, respectively.

**Figure 4:**
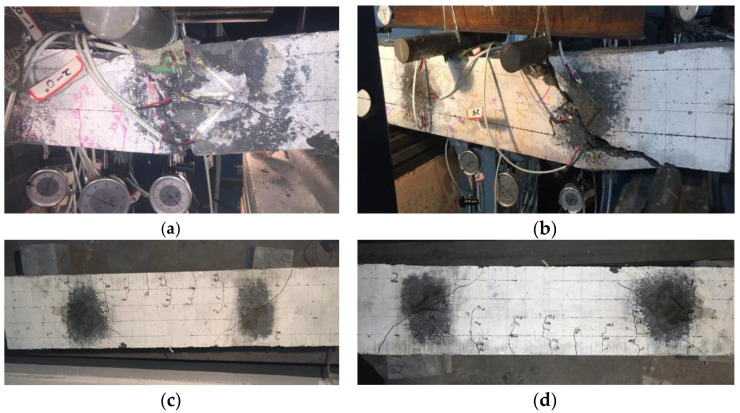
Failure modes of the recycled concrete beams. (**a**) B-740 failure mode. (**b**) B-1010 failure mode. (**c**) B-1280 failure mode. (**d**) B-1550 failure mode.

By static loading, the final failure modes of these four recycled concrete beams are shown in Figure 4. The main data obtained from the experiment are listed in Table 6 and Table 7, respectively. In Table 7, the shear fracture strength and shear strength are the ratio of P_cr_ to b × h and P_u_ to b × h.

### 2.3. The Calculation Formulas for Shear Capacity

As stated previously, shear capacity is an important parameter in the design of the beam-type structure. For concrete beams, the shear force is mainly borne by the concrete and stirrups. In this section, several computation models for shear capacity of the concrete beam without stirrups are briefly reviewed.

#### 2.3.1. ACI Computation Formula (ACI 318M-14) 

The computation formula of shear capacity in ACI code (ACI 318M-14) [31] is obtained according to the test of small volume concrete beams. These formulas do not take into account the size effect. The computation equations are:

(1) Vc=(0.16fc′+17ριad)bd≤0.29fc′bd

where a = shear span length of beam (m), d = effective height; b = web width, $\rho_{\iota }$ = longitudinal reinforcement ratio, $f_{c}^{\prime }$ = compressive strength of concrete.

#### 2.3.2. EN 1992-1-1 

The shear capacity in EN 1992-1-1 [32] is calculated using the following formula:

(2) Vc=(0.18κ(100ριfc′)13)bd≥(0.035κ32fc′)bd

where $\kappa =1+\sqrt{\frac{200}{d}}\leq 2.0$, d = effective height; b  = web width, $\rho_{\iota }$ = longitudinal reinforcement ratio, $f_{c}^{\prime }$ = compressive strength of concrete.

#### 2.3.3. GB50010-2010 (China)

The computation formula of shear capacity in China’s current norms “concrete structure design specifications” (GB50010-2010) [33] is:

(3) $$V_{c}=\frac{1.75}{\lambda +1}f_{t}\times b\times h_{0}$$

where λ is the shear span ratio of the calculated section, λ = 1.5 if λ≤1.5, λ = 3 if λ≥ 3; $\;f_{t}$ is the design value of concrete axial tensile strength, *b* is the section width, $\;h_{0}$ is the effective height.

#### 2.3.4. Computation Formula by Truss-Arch Model

The computation formula of shear capacity based on Truss-arch Model in reference [34] is:

(4) $$V_{c}=0.55\left(\sqrt{\lambda^{2}+1}-\lambda \right)f_{c}bh_{0}$$

where λ = 1.5 if λ ≤ 1.5, λ = 3 if λ  ≥ 3, $h_{0}$ = effective height; *b* = web width, $f_{c}$ = compressive strength of concrete.

#### 2.3.5. Computation Formula of Reference

The shear capacity formula proposed by Wang et al. [15] for concrete beams is:

(5) $$V_{c}=\left(1.2-0.2a\right)\varphi \times f_{c}^{\prime }\times b_{w}\times d_{v},\;\;\varphi =\frac{2B+A\rho_{s}f_{s}/f_{c}^{\prime }}{2B\lambda f_{c}^{\prime }/\rho_{s}f_{s}+1}$$

where a = shear span length of beam (m), *A* = 0.0837, *B* = 1.075,  λ is the shear span ratio of the calculated section, λ = 1.5 if λ ≤ 1.5, λ = 3 if λ ≥ 3, $d_{v}\;$ = effective height; $b_{w}\;$ = web width, $\rho_{s}\;$ = longitudinal reinforcement ratio, $f_{s\;}$ = yield strength of steel, $\;f_{c}^{\prime }$ = compressive strength of concrete. The size effect is considered in the above formula.

#### 2.3.6. Computation Formula Proposed by Bažant

Bažant [16] applies the size effect law to the computation of shear capacity of reinforced concrete beams without web reinforcement. The computation formula is as follows:

(6) Vc=μρw38bw(1+da)fc′d0d1+d0d,d0=kf0′−23

where $k=3800\sqrt{d_{a}}$ if $d_{a}\;$ is known, k=3330  if not, μ = 13.3 for best fit, μ = 10 for design, $d_{a}$ = aggregate size, $\rho_{w}$ = longitudinal reinforcement ratio, $\frac{a}{d}$ = Shear-span ratio, $f_{c}^{\prime }$ = compressive strength of concrete.

#### 2.3.7. Computation Formula Based on Modified Pressure Field Theory

Using the modified pressure field theory, the computation formula of shear capacity of reinforced concrete beams based on size effect is derived by Zhou in the reference [35] as:

(7) $$V_{c}=\beta \sqrt{f_{c}^{\prime }}b_{w}d_{v}\frac{1+\sqrt{\frac{0.24}{d_{a}}}}{\sqrt{1+\frac{d}{\lambda_{0}d_{a}}}}$$

where $\lambda_{0}$ = 25, $d_{a}$ = aggregate size, $\rho_{w}$ = longitudinal reinforcement ratio, $d_{v}$ = effective height; $b_{w}$ = web width, $f_{c}^{\prime }$ = compressive strength of concrete. β can be calculated as [36]:

(8) $$\beta =\frac{0.4}{\sqrt{1+6.2\varepsilon_{sx}d_{v}}}$$

where $\varepsilon_{sx}=\frac{\frac{M}{d_{v}}+V}{E_{s}A_{S}},d_{v}=0.9d$, *M*, *V* is the bending moment and shear force of the calculated section.

#### 2.3.8. Computation Formula Proposed by Luo

Combined with Bažant size effect law, Luo [19] derived the shear strength formula of reinforced concrete beams without web reinforcement. The formula can be applicable to both diagonal-tension failure and shear-compression failure.

(9) $$V_{c}=0.145\frac{c_{0}f_{c}^{\prime }b\frac{2d+3c_{0}}{a_{0}}}{\sqrt{1+\frac{d}{d_{0}}}}$$

(10) $$c_{0}=\{\begin{array}{c} 30{f_{c}^{\prime }}^{-0.5}\rho^{0.6}d{\left(\frac{a}{d}\right)}^{-1},\;1\leq \frac{a}{d}<3\\ 10{f_{c}^{\prime }}^{-0.5}\rho^{0.6}d,\;3\leq \frac{a}{d} \end{array}$$

(11) a0=3.3(ρ(da)2(1−ρ)2)13a

where $d_{a}$ = aggregate size, ρ = longitudinal reinforcement ratio, $\frac{a}{d}$ = Shear-span ratio, $f_{c}^{\prime }$ = compressive strength of concrete. a = shear span length of beam.

#### 2.3.9. Computation Formula Based on Fracture Mechanics

Zsutty [28] used the fracture mechanics method to predict the shear capacity of reinforced concrete beams without stirrups. Based on statistical analysis, the following equation is proposed:

(12) Vc=2.21(fc′ριda)13bd

where a = shear span length of beam (m), d = effective height; b = web width, $\rho_{\iota }$ = longitudinal reinforcement ratio, $f_{c}^{\prime }$ = compressive strength of concrete.

## 3. Analysis and Comparison of Experimental Results

From Table 6 and Table 7, the maximum shear stress, shear fracture strength, and shear strength of the recycled concrete beam all have obvious size effect. Generally, there is a law that the maximum shear stress, shear fracture strength, and shear strength decrease with the increase in beam section height and beam length. However, the shear strength of B-1550 is abnormal. The possible causes of this exception are: (1) the restraint effect of concrete cover for B-1550 is stronger than that of other beams; (2) The judgment of ultimate failure state is different from that of other specimens; (3) B-1550 was tested two months later than other beams. The shear strength of recycled aggregate concrete beam may increase over time.

**Figure 5:**
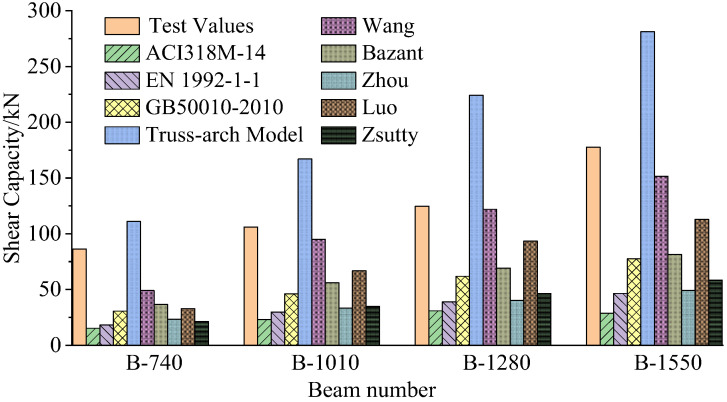
Comparisons of the shear capacity test values and theoretical values.

Next, the applicability of shear capacity formulas in Section 2.3 is studied for these recycled concrete beams. For ease of comparison studies, the experimental data and the calculated values of the shear capacities are all listed in Table 8. Note that the data in brackets in Table 8 represent the relative errors between the test values and the theoretical calculation values. For intuitive analysis, Figure 5 presents the test values and calculated values in Table 8 for B-740, B-1010, B-1280 and B-1550, respectively. From Table 8 and Table 9 presents the safety reserve coefficients (the ratios of test values to calculated values) obtained by various shear capacity formulas. 

In Table 9, the data less than 1 indicates insecurity and the data greater than 1 indicates conservatism. Overall, the safety reserve coefficients in Table 9 for each theoretical formula decrease with the increase in beam section height and beam length. This trend is consistent with the size effect law of shear strength from Table 7. Note that the safety reserve coefficients of B-1550 increase abnormally for most theoretical formulas since the tested shear capacity of B-1550 is abnormally large, as stated previously. It was found from Table 9 that only the computation results obtained by the truss-arch model are unsafe, since all the safety reserve coefficients for this model are less than 1. This means that the truss-arch model can not be used for calculating the shear capacity of the recycled concrete beam. The reasons for the unsafe calculation results of the truss-arch model may lie in the following aspects: (1) Truss-arch model is more suitable for beams with stirrups since the stirrups play an important role in forming shear capacity. However, the beam specimens in this work are not equipped with stirrups. (2) Compared with ordinary concrete, recycled concrete is easier to crack and more unfavorable to the formation of truss-arch bearing model. According to the data by ACI 318M-14, EN 1992-1-1 and GB50010-2010, the safety reserve coefficient of ACI 318M-14 is the highest, followed by EN 1992-1-1 and GB50010-2010. From the perspective of engineering application, the computation formula of Chinese code for shear capacity is more appropriate. The computation results by Zhou’s formula and Zsutty’s formula are also too conservative and uneconomical for engineering construction. The reasons for the too conservative calculation results are as follows: The sizes of the beam specimens in this work are relatively small compared with the actual beams in engineering. According to the general law of size effect, the smaller the size, the higher the shear strength of the beam. This leads to the shear capacity calculated by the theoretical formulas is much smaller than the test value since these theoretical formulas do not consider the size effect well. Thus, the calculation results are all too conservative. In comparison, the size effect is considered to some extent in Bazant’s, Wang’s, and Luo’s formulas, so the corresponding calculated results have little deviation from the measured values. From Table 9, the calculated results by Wang’s formula are closest to the measured values of shear capacity. However, the corresponding safety reserve coefficient is too low and the potential safety hazard is large. The results obtained by Luo’s formula are also consistent with the experimental results and have the appropriate safety reserve coefficients. Thus, Luo’s formula can be recommended to calculate the shear capacity of recycled concrete beams in engineering practice.

Finally, the research results of this work are briefly compared with those of some similar studies in references [24,27,37,38,39]. The results of reference [27] show that: (1) the ACI and CSA codes methods are applicable to the shear capacity of recycled concrete beams, provided the equivalent mortar volume (EMV) method of mix design is used; (2) The shear resistance of the reinforced recycled concrete beams had a tendency to increase with decrease in the overall depth of the beam. Note that the length range of the beams in their experiment is between 2.1 m and 3.7 m, and stirrups are configured in the beams. The size effect observed in reference [27] is similar to that in this work. In reference [37], the results of five recycled concrete beams (2000 mm × 150 mm × 300 mm) without stirrups indicate that the safety reserve coefficients obtained by GB50010-2010 for these beam specimens are around 1.9~2.1. This is close to the calculation results shown in Table 9 of this work. In reference [24], the results of nine beam specimens (2300 mm × 150 mm × 300 mm) with stirrups show that GB50010-2010 is applicable to the shear capacity calculation. The replacement rates of recycled aggregate are 25%, 50%, and 75%, respectively. The calculated safety reserve coefficients for these specimens are around 1.1~1.3. In reference [38], the safety reserve coefficients obtained by GB50010-2010 for six reinforced recycled concrete beams (2300 mm × 200 mm × 300 mm), with 30%, 50%, 80%, and 100% recycled aggregate replacement rate, are also around 1.1~1.3. It is also observed that the safety reserve coefficient decreases with the increase in the replacement rate of recycled aggregate. Compared with the results of this paper, the results of references [24,38] indicate that: (1) Stirrups play an important role in the shear capacity of beams. The shear performance of recycled concrete beams with stirrups is close to that of ordinary concrete beams; (2) The safety reserve coefficient may have a tendency to decrease with increase in the beam length and recycled aggregate replacement rate. Based on experimental data collected from 22 literature, the research results of reference [39] show that the ACI code, GB50010-2010, and Zsutty’ formula are not ideal for the shear capacity calculation of recycled concrete beams. This is consistent with the above analysis of the results in this work.

## 4. Conclusions

In this work, four specimens with different sizes were fabricated and tested to investigate the shear capacity of the recycled concrete beam without web reinforcement. The shear behavior of recycled concrete beams is studied by the bending test. According to the experimental and theoretical research, the following conclusions can be drawn as: (1) The maximum shear stress, shear fracture strength, and shear strength of the recycled concrete beam all have obvious size effects. Generally, there is a law that the maximum shear stress, shear fracture strength, and shear strength decrease with the increase in beam section height and beam length. (2) The shear failure mechanism of recycled concrete beam is similar to that of ordinary concrete beam. At present, the concrete design codes of various countries can be used to calculate the shear capacity of recycled concrete beams. The Chinese code is in the best agreement with the test results. (3) The existing computation methods can only be used to estimate the shear capacity of small-size recycled concrete beams. For large recycled concrete beams, the size effect should be considered and the existing method should be modified to obtain a more accurate estimation of shear capacity. (4) The comparative study shows that Luo’s formula is most suitable for the shear capacity computation of the recycled concrete beam. It has been shown that it is very necessary to consider the size effect in the computation of shear capacity for the recycled concrete beams. Note that there are some issues that need to be further studied in the future. First, the shear capacity of beams with different replacement rates of recycled aggregate needs to be further studied since only 100% replacement rate is considered in this work. Second, the size effect on shear capacity of recycled concrete beams with stirrups should be further studied for engineering application. Third, the applicability of various shear capacity calculation formulas should be further verified by more experiment data of recycled concrete beams with larger sizes.

## Figures and Tables

**Table 1:** Tested parameters of fine aggregate.

Fine Aggregate	Grain Diameter (mm)	Apparent Density (kg/m^3^)	Bulk Density (kg/m^3^)	Water Ratio (%)	Modulus of Fineness	Water Absorption (%)
River sand	<5	2548	1211	6.8	1.83	2.9

**Table 2:** Tested parameters of recycled coarse aggregate.

Apparent Density (kg/m^3^)	Bulk Density (kg/m^3^)	Water Absorption (%)	Water Ratio (%)	Crush Value Index (%)	Cavity Ratio (%)	Porosity (%)	Incubation Rate (%)
2481	1240	6.3	2.2	19.9	5.7	51.0	42.9

**Table 3:** Tested parameters of the reinforcement bar.

Reinforcement Type	Diameter/mm	Yield Strength f_y_/MPa	Tensile Strength f_u_/MPa	Elastic Modulus E_s_/MPa
HRB400	10	481	603	1.96 × 10^5^
HRB400	14	459	528	1.89 × 10^5^
HRB400	16	421	582	2.12 × 10^5^
HRB400	18	414	543	2.21 × 10^5^

**Table 4:** Mix proportion design of recycled aggregate concrete.

Replacement Rate of Coarse Aggregate	Water Cement Ratio	The Quantities of Cement, Water, Sand, and Coarse Aggregate			
100%	1:0.34	Cement	Water	Sand	Coarse aggregate
		623.3 kg/m^3^	211.8 kg/m^3^	491.4 kg/m^3^	1073.5 kg/m^3^

**Table 5:** Beam size and reinforcement design.

Dimensions: L × b × h (mm)	Clear Span	Reinforcement Design (mm)	Thickness of Reinforcement Cover (mm)
740 × 120 × 120	540 mm	2φ10	10
1010 × 120 × 180	810 mm	2φ14	15
1280 × 120 × 240	1080 mm	2φ16	20
1550 × 120 × 300	1350 mm	2φ18	25

**Table 6:** Maximum shear stress of each beam.

Beam Number	Load (kN)	*τ*_max_ (MPa)
B-740	7.6	0.858
B-1010	10.9	0.453
B-1280	6.6	0.293
B-1550	6.1	0.245

**Table 7:** Midspan deflection, shear capacity and shear strength of each beam.

Beam Number	Cracking Load P_cr_ (kN)	Shear Fracture Strength V_cr_ (MPa)	Ultimate Load P_u_ (kN)	Shear Strength V_u_ (MPa)	Mid-Span Deflection △ (mm)
B-740	7.8	0.619	86.3	6.84	2.7
B-1010	11.3	0.596	106	5.59	4.08
B-1280	7.05	0.277	124.7	4.9	3.19
B-1550	7.1	0.222	177.7	5.56	5.01

**Table 8:** Shear capacities obtained by the experiment and theoretical formulas (kN).

Beam Number	B-740	B-1010	B-1280	B-1550
Test values	86.3	106.0	124.7	177.7
ACI [31]	15.3	23.0	30.9	28.7
	(82%) *	(78%)	(75%)	(84%)
EN 1992-1-1 [32]	18.1	29.8	39.0	46.5
	(79%)	(72%)	(69%)	(74%)
GB50010-2010 [33]	30.6	46.1	61.8	77.5
	(65%)	(57%)	(50%)	(56%)
Truss-arch Model [34]	111.0	167.1	224.2	281.2
	(−29%)	(−58%)	(−80%)	(−37%)
Wang [15]	49.1	95.1	122.0	151.6
	(43%)	(10%)	(2%)	(15%)
Bazant [16]	36.6	56.1	69.2	81.5
	(58%)	(47%)	(45%)	(54%)
Zhou [35]	23.3	33.2	40.3	49.1
	(73%)	(69%)	(68%)	(72%)
Luo [19]	32.9	66.8	93.5	112.9
	(62%)	(37%)	(25%)	(36%)
Zsutty [28]	21.2	34.9	46.4	58.4
	(75%)	(67%)	(63%)	(67%)

* The data in brackets represent the relative errors between the test and theoretical values.

**Table 9:** The safety reserve coefficients obtained by various shear capacity formulas.

Beam Number	B-740	B-1010	B-1280	B-1550
ACI [31]	5.5	4.6	4	4
EN 1992-1-1 [32]	4.6	3.6	3.2	3.8
GB50010-2010 [33]	2.7	2.3	2	2.3
Truss-arch Model [34]	0.8	0.6	0.6	0.6
Wang [15]	1.7	1.1	1	1.1
Bazant [16]	2.3	1.9	1.8	2.2
Zhou [35]	3.6	3.2	3.1	3.6
Luo [19]	2.5	1.6	1.3	1.6
Zsutty [28]	3.6	3.1	2.7	3

## Data Availability

Not applicable.
